# Editorial: White matter dementia: neuropathological and neuropsychological underpinnings and state of the art diagnosis methods and treatments

**DOI:** 10.3389/fneur.2023.1268295

**Published:** 2023-08-17

**Authors:** Christopher M. Filley, Iris-Katharina Penner, Lisa Schäfer, Wolfgang Köhler

**Affiliations:** ^1^Behavioral Neurology Section, Departments of Neurology and Psychiatry, Marcus Institute for Brain Health, University of Colorado School of Medicine, Aurora, CO, United States; ^2^Department of Neurology, Inselspital, Bern University Hospital, University of Bern, Bern, Switzerland; ^3^Department of Neurology, Leukodystrophy Clinic, University of Leipzig Medical Center, Leipzig, Germany

**Keywords:** white matter, dementia, Alzheimer's disease, traumatic brain injury, vascular dementia

The higher functions of humans are widely accepted as arising from the activity of brain neurons, and neuroscience has traditionally concentrated on neuronal components within the gray matter, particularly of the cerebral cortex. Whereas, a primary focus on cortical cell bodies, dendrites, and synapses has proven enormously successful, a corticocentric approach neglects the contributions of subcortical regions (1), and white matter is easily the most prominent subcortical structure. In this context, a dementia syndrome arising from selective white matter pathology was proposed in 1988 (2), based primarily on study of toluene abuse demonstrating striking white matter toxicity that produced dementia (3). Over the ensuing years, the idea of white matter dementia steadily gained credibility as transdiagnostic synthesis of many disorders disclosed numerous commonalities implicating a critical role of white matter connectivity in human behavior and its disorders. The many areas of neurology relevant to white matter dementia are exemplified by key studies of white matter pathology ranging from genetic diseases such as adrenoleukodystrophy (4) to vascular diseases including stroke (5). To recognize progress in this field, an invited international Research Topic of scholarly papers dedicated to white matter dementia now appears in *Frontiers in Neurology*. The idea of white matter dementia has indeed come of age.

The Research Topic begins with two papers on genetic diseases. Lotz-Havla et al. investigate blood biomarkers in phenylketonuria, in which early and continuously treated patients continue to manifest neurobehavioral features with MRI white matter changes. This pilot study concludes that while glial fibrillary acidic protein and neurofilament light are not elevated, larger studies are warranted to investigate these biomarkers because even treated patients may harbor increased risk for neurodegenerative diseases (Lotz-Havla et al.). Delving further into treatment, Bergner et al. present two patients with CSF1-receptor-related leukoencephalopathy who received hematopoietic stem cell treatment and, after 6 months, showed improvement of white matter dementia that is not expected in untreated disease. This study also found that myelin imaging using (18F) florbetaben with positron emission tomography suggested post-treatment stabilization of white matter degeneration (Bergner et al.).

Turning to vascular disease, Pansieri et al. offer a thorough review of vascular dysfunction in white matter dementia. Combining data from vascular dementia, Alzheimer's disease (AD), and multiple sclerosis (MS), the authors eloquently describe a shared landscape of vascular disease that underscores the importance of adequate blood supply to the aging brain (Pansieri et al.). On a related topic, Yu et al. present data from a study of white matter hyperintensities in demented adults showing that lesions within cholinergic pathways exert a greater impact on dementia severity among APOE ε4 carriers than non-carriers. This effect may result from the impact of altered lipid homeostasis and inflammation related to APOE ε4 that may exacerbate vascular injury within cholinergic tracts.

Two of the most familiar disorders with prominent white matter pathology are MS and traumatic brain injury (TBI). Hebert and Filley present a review of how multifocal or diffuse white matter lesions in these disorders impact multisensory integration and cognitive function. Adding to an emerging area of behavioral neurology, this paper integrates sensory with cognitive dysfunction by highlighting the propensity of white matter lesions – from either demyelination or diffuse axonal injury – to damage cognitively relevant regions, or, alternatively, affect visual, auditory, or vestibular systems to the extent that engagement of normal cognitive regions is overwhelmed (Hebert and Filley).

White matter clearly undergoes developmental alterations, and Mendez Colmenares et al. address changes in aging. Using a novel imaging analysis that includes four diffusion tensor imaging (DTI) parameters – fractional anisotropy, mean diffusivity, radial diffusivity, and axial diffusivity – these investigators found correlations with processing speed and executive function in cognitively healthy older adults that were not evident using individual DTI parameters (Mendez Colmenares et al.). These observations may assist in the classification of white matter disorders, and in understanding the neurobiology of aging and dementia (Mendez Colmenares et al.).

Returning to neurodegenerative disease, Holden et al. present new data on diminished posterior white matter integrity and symptoms relevant to higher visual dysfunction in posterior cortical atrophy (PCA). Using the Colorado Posterior Cortical Questionnaire (CPC-Q), these investigators report that higher visual symptoms of PCA are strongly correlated with microstructural alterations in posterior white matter. Since PCA usually features AD pathology, these findings may be relevant to AD pathogenesis, and in clinical practice, the CPC-Q may serve as a convenient tool with which to assess disrupted posterior tracts in a variety of white matter disorders (Holden et al.).

Last, drawing on extensive experience proposing and promoting the idea of white matter dementia, Filley presents a thorough and updated overview of the concept from its inception to the present. Among the many advances in the field has been an exciting focus on the prevention and treatment of white matter pathologies, which may have transformative implications for a wide variety of hitherto poorly understood and irreversible disorders. In its broadest sense, white matter dementia contributes to a fundamental goal of behavioral neurology, the understanding of brain-behavior relationships.

In summary, this Research Topic presents a group of papers that highlight white matter dementia. Genetic diseases, vascular disorders, MS, and TBI all serve as informative examples of white matter pathology with neurobehavioral implications, and new insights are offered on white matter in aging and degenerative disease. White matter dementia has become an important concept that calls attention to white matter disorders and cognition, but investigation of human behavior in its entirety is clearly including white matter within its scope. This development adds a much needed complement to the traditional corticocentric emphasis of neuroscience. Indeed, contrary to long-held opinion that gray matter, mainly of the cerebral cortex, is most crucial for understanding human behavior, recent evidence indicates that pathology in white matter may even be more critical for producing neurobehavioral impairment (6). These insights promise to hasten the study of white matter with respect to cognition and emotion, potentially leading to deeper understanding of prevalent disorders such as AD that are as scientifically challenging as they are disabling. Moat fundamentally, white matter dementia adds to basic knowledge of how the brain mediates human behavior.

## Author contributions

CF: Conceptualization, Data curation, Formal analysis, Investigation, Supervision, Writing—original draft. I-KP: Conceptualization, Data curation, Writing—review and editing. LS: Conceptualization, Data curation, Writing—review and editing. WK: Conceptualization, Data curation, Formal analysis, Supervision, Writing—review and editing.

## References

[1] Parvizi J. Corticocentric myopia: old bias in new cognitive sciences. Trends Cogn Sci. (2009) 13:354–9. 10.1016/j.tics.2009.04.008 19595625

[2] Filley CM Franklin G M Heaton RK Rosenberg NL. White matter dementia: Clinical disorders and implications. Neuropsychiatry Neuropsychol Behav Neurol. (1988) 1:239–54.

[3] Filley CM Kleinschmidt-DeMasters BK. Toxic leukoencephalopathy. N Engl J Med. (2001) 345:425–32. 10.1056/NEJM200108093450606 11496854

[4] Schäfer L Roicke H Fischer M Sühnel A Köhler W. Cognitive functions in adult-onset phenotypes of X-linked adenoleukodystrophy. Ann Neurol. (2021) 90:266–73. 10.1002/ana.26141 34105176

[5] Goldberg MP Ransom BR. New light on white matter. Stroke. (2003) 34:330–2. 10.1161/01.STR.0000054048.22626.B9 12574526

[6] Reber J Hwang K Bowren M Bruss J Mukherjee P Tranel D. Cognitive impairment after focal brain lesions is better predicted by damage to structural than functional network hubs. Proc Natl Acad Sci U S A. (2021) 118:e2018784118. 10.1073/pnas.2018784118 33941692 PMC8126860

